# Establishment of a detection assay for DNA endonuclease activity and its application in the screening and prognosis of malignant lymphoma

**DOI:** 10.1186/s12858-018-0096-6

**Published:** 2018-07-31

**Authors:** Renquan Lu, Yingchao Wang, Xiaofeng Xu, Suhong Xie, Yanchun Wang, Ailing Zhong, Hui Zheng, Yiwen Yu, Xiang Gao, Lin Guo

**Affiliations:** 1 Department of Clinical Laboratory, Fudan University Shanghai Cancer Center, 270 DongAn Road, Xuhui District, Shanghai, 200032 China; 2 Department of Clinical Laboratory, Shanghai Proton and Heavy Ion Center, Shanghai, 201321 China

**Keywords:** Neoplasm, Endonuclease, Lymphoma, Prognosis, Tumor marker

## Abstract

**Background:**

Endonucleases play critical roles in maintaining genomic stability and regulating cell growth. The purpose of this study was to evaluate detection of endonuclease activity as an indicator in the early diagnosis and prognosis of lymphoma.

**Results:**

The method of endonuclease activity determination was successfully established and applied to compare cancer patient and control cohorts. Endonuclease activities of cancer tissues were significantly higher than those of adjacent control tissues (*P* < 0.001). We next investigated endonuclease activity in peripheral blood of enrolled patients and the controls, which were also significantly higher in patients than in controls (*P* = 0.015). Additionally, endonuclease activities were elevated in the metastasis subgroup compared with the non-metastasis subgroup(*P* = 0.038), whereas no significant difference was found between age(≤ 56y, > 56y) and gender (*P* = 0.736 > 0.05 and *P* = 0.635 > 0.05, respectively). Although there was no significant difference between control group with the non-metastatic cancer patients (*P* = 0.800 > 0.05), endonuclease activities were lower in the control group compared with the non-metastatic cancer patients with lymphoma (*P* = 0.033). The progression-free survival probability of patients with elevated R ratios(R ratio ≥ 1.4) was significantly lower than that of patients with lower R ratios (R ratio < 1.4).

**Conclusions:**

An assay was established to detect the endonuclease activity,which might be useful for the prognosis of cancers, especially lymphoma.

## Background

Endonucleasesare nucleic acid hydrolases that hydrolyze internal phosphodiester bonds of molecular chains to generate oligonucleotides. They play an important role in multiple DNA metabolic processes including the removal of RNA primers in delayed chain replication, long patch base excision repair (LP-BER), telomere stability, and elimination of apoptotic DNA fragments [1–6]. Different gene expression patterns determines the identity of each cells. The occurrence and development of tumors can be seen as a microcosm in the evolutionary process. One of their sign is the accumulation of many dysfunctional genes caused by gene mutation, structural transfer, chromosome deletion, microsatellite expansion and contraction [7].Numerous studies have confirmed that genomic instability plays a major role in the development of tumors. Aberrant gene causes cancer and genetic disease. Changes in endonuclease activity are likely to be associated with the development and progression of neoplasms, while the enzyme plays a key role in maintaining genome integrity.

There are many reports about the relationship between cancer and endonucleases including apurinic/apyrimidinic endonuclease 1 (APE1), Flap endonuclease-1 (FEN1) and Dicer. Accumulating evidence indicates that altered Human APE1 expression patterns are associated with carcinogen susceptibility and cancer development or progression. APE1 showed high expression in a variety of cancers including germ cell tumors, gliomas, rhabdomyosarcoma, breast, liver, non-small cell lung cancer and ovarian cancer [8]. FEN1 is a key factor during the maintenance of genomic stability and protection against tumorigenesis [9, 10]. Overexpression of Dicer predicts poor survival of colorectal cancer patients, which is in line with an investigation of prostate cancer [11]. Repeat instability of gene promoters and associated differential gene expression might play a major role in tumors [12]. These studies have provided the experimental basis for the current study that aimed to explore the relationship between peripheral blood and tumor nuclease activities.

Lymphomas, defined as non-Hodgkin’s, Hodgkin’s, and chronic lymphocytic leukemia /small lymphocytic lymphomas, are the most common hematological malignancies in western countries with an estimated 95,520 newly diagnosed cases each year in the United States [13]. Although the incidence of lymphomas is not in the top 10 in China according to the 2012 report of the malignant tumor registration collected from National Cancer Registry in 2015, its incidence in males is ranked in the top 10. Lymphoma was the 10th most frequent cause of cancer death in China with estimated deaths of 43,000 in 2012 [14]. The incidence rate of lymphoma was 6.48 / 100,000 in 1998–2010, which was increased from 3.78 / 100,000 in 1998 to 8.88 / 100,000 in 2010 with an increasing rate of 136.17% (*P* < 0.05) [15]. Studies have shown that the number of lymphoma cases is growing in China. At present, lymphoma diagnosis should be combined with the clinical manifestations of patients, physical examination, laboratory tests, and imaging and pathological findings. It is desirable to determine endonuclease activity as an experimental basis for screening markers of lymphoma. In the majority of previous studies, tissue samples were used to identify the role of endonucleases in cancer occurrence. Therefore, the above methods are traumatic. To minimize-trauma, we established a detection assay in this study using peripheral blood samples. Such peripheral blood samples are convenient to be used in follow-up disease-monitoring. Furthermore, we elucidated the relationship between endonuclease activity and neoplasm progression.

## Methods

### Patients and specimens

Peripheral blood samples (*n* = 179) and tissue samples including cancer tissue and matched adjacent tissue from October 2014 to August 2016 were used in this study. Tissue samples were surgical specimens obtained from ovarian cancer patients. All subjects, enrolled in this study at the Fudan University Shanghai Cancer Center (Shanghai, China) provided written informed consent. The Institutional Ethics Committee approved the study protocol according to the guidelines of Helsinki conventions. We only collected tissue samples from seven cases for preliminary-experimentation. Peripheral blood samples were anticoagulated by EDTA. We first collected 61 normal human peripheral blood samples and then chose 48 samples with normal hepatic functions, meeting the requirements for the control group. Through retrospective analysis, 131 patients with malignant lymphoma (*n* = 44), gastrointestinal cancer (*n* = 15), lung cancer (*n* = 50), and other neoplasm (*n* = 22) met the requirements for the malignant tumor group.

Human ovarian cancer cell lines SKOV3, HO8910, GM0369, and HosEpic were acquired from the Chinese Academy of Sciences (Shanghai, China). Lymphoma cell lines Pfeiffer (ATCC® CRL-2632™) and SU-DHL-4 (ATCC® CRL-2957™) were acquired from the American Type Culture Collection.

### Establishment of the endonuclease activity detection method

Cells were obtained from suspensions of tissue sample dissociated by a high speed homogenizer. Cultured cells were removed from dishes by washing with PBS. Mononuclear cells were isolated from human peripheral blood samples through human whole blood mononuclear cell separation fluid. Erythrocyte lysis buffer was used to exclude erythrocytes from the selected cells. Chaps lysis buffer was used to lyse cells on ice for 60 min. The supernatants of lysates were collected and stored at − 80 °C until use. Finally, endonuclease activity was determined by the cleaved degree of the plasmid *pwpxl* through agarose gel electrophoresis.

### Verification of the established method

The feasibility of using agarose gel electrophoresis to detect endonuclease activity was demonstrated by verifying the number of bacterial clones. For agarose gel electrophoresis and counting bacterial clones, 200 ng *pwpxl* was added to 8.1 μl lysis supernatant of collected ovarian cancer cell line HO8910. The total volume of the mixture was 10 μl. The mixture was incubated at 37 °C for 30 min and then mixed with 2 μl 6 × loading buffer before agarose gel electrophoresis. For the colony count of bacterial clones, a 1 / 3 mixture (3.33 μl) was extracted and added to 30 μl competent cells. The mixture was then placed in an ice bath for 30 min, followed by heating it at 42 °C for 90 s. Then the mixture was combined with 300 μl LB (−) culture medium and shaken for 45 min. Finally, the mixture was centrifuged at 1500×*g* for 3 min, 200 μl supernatant was discarded, and the remaining supernatant was applied to a plate. The number of colonies was counted after incubation at 37 °C for 16 h.

### Optimization of the enzymatic activity detection method

By varying incubation times while maintaining other experimental conditions, we explored the optimal incubation time. After several experiments, the optimal ratio of lysate from blood samples was determined. The relationship between the degree of plasmid cleavage and total protein was determined by agarose gel electrophoresis.

### Calculation of the R ratio

We used Image J software to scan the brightness of each band to obtain the R overall brightness, R above brightness, and R below brightness. The calculation formula was as follows: R1 sample = (R overall brightness -R above brightness -R below brightness) ÷ (R above brightness + R below brightness). To reduce or eliminate the difference in brightness, we used the following formula: R2 sample = R1 sample ÷ R overall brightness of the negative control. To reduce or eliminate differences between experiments, we selected the same specimen from different experiments. Then, the calibration coefficient was obtained and multiplied to calculate the R ratio.

### Statistical analysis

The difference in endonuclease activities of normal control and malignant tumor groups was analyzed by one-way analysis of variance, and the least squares difference test. Progression-free survival (PFS) time was calculated by the Kaplan–Meier method. In all analyses, *P*-values of less than 0.05 were considered to be statistically significant. All statistical calculations were performed with SPSS 18.0.

## Results

### Feasibility of using agarose gel electrophoresis to detect endonuclease activity

**Fig. 1:**
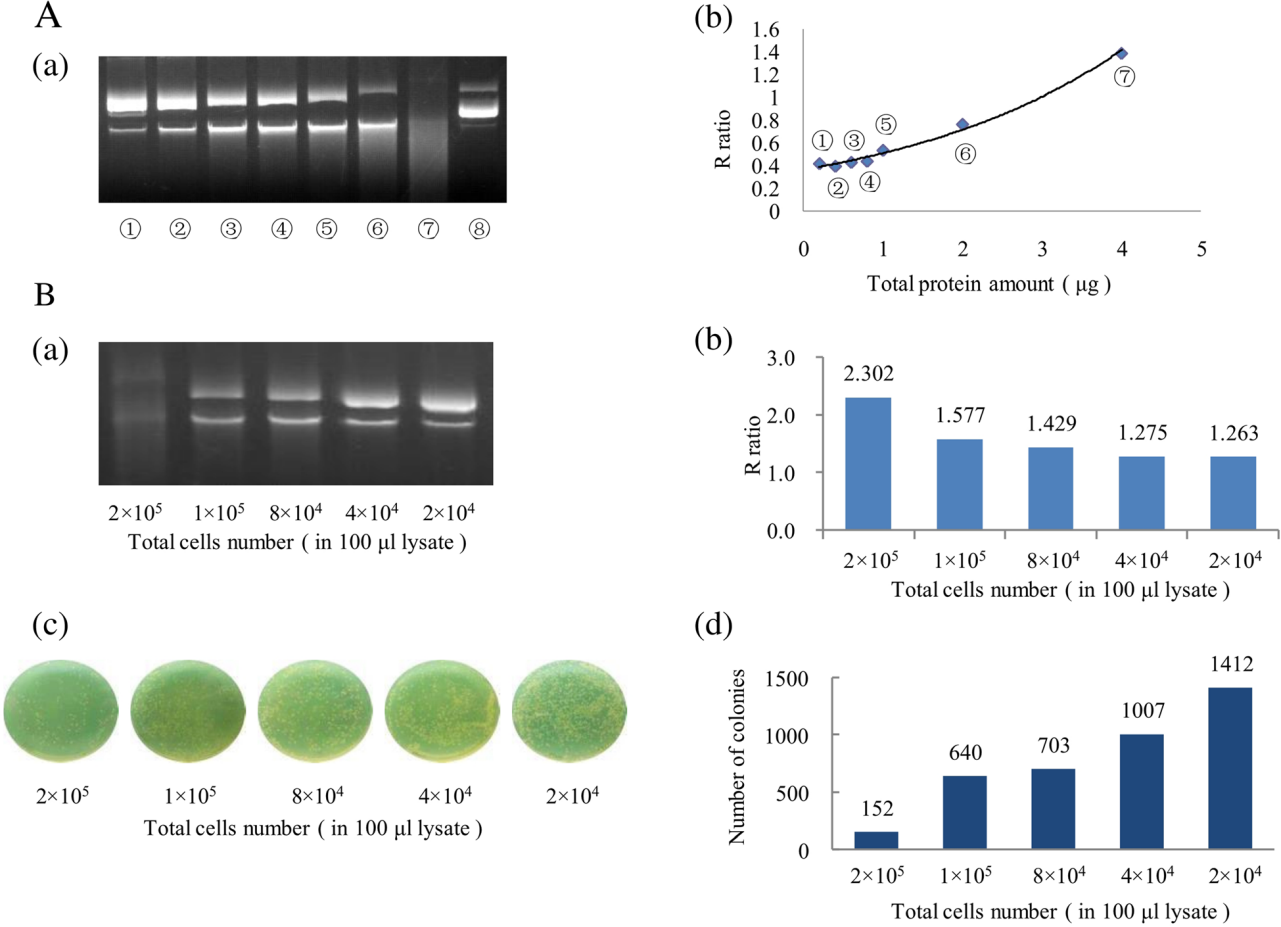
Validation of the feasibility of agarose gel electrophoresis to detect endonuclease activity. **A** (a) Agarose gel electrophoresis of HO8910 cell lysates with different total protein amounts.: 0.2 μg (1),: 0.4 μg (2),: 0.6 μg (3),: 0.8 μg (4),: 1 μg (5),: 2 μg (6),: 4 μg (7),: 0 μg (8). (b) Curve of the total protein amount (y) and R ratio(x). y = 0.289 x + 0.210, R^2^ = 0.996. **B** (a) Agarose gel electrophoresis of 8910 cells at different total numbers in 100 μl Chaps lysis buffer. (b) Histogram of R ratios of HO8910 cells. (c) HO8910 cell’s bacterial colony number. (d) Histogram of HO8910 cell’s bacterial colony number

A total of 200 ng *pwpxl* was digested by HO8910 cell lysate with different total protein amounts(0.2 μg, 0.4 μg, 0.6 μg, 0.8 μg, 1 μg, 2 μg, 4 μg, and 0 μg) and subjected to agarose gel electrophoresis (Fig. 1A a). Using Image J software and the formula described above, R ratios were calculated for HO8910 cell lysates with different amounts of total protein. For lysates of the same cell line, the lower the total protein content, the lower the R ratio and degree of plasmid cleavage, suggesting a positive correlation between the protein concentration and enzymatic activity. When the total protein amount ranged from 0.8 to 4 μg, there was a linear relationship between the R ratio and total protein quantity, y = 0.289 x + 0.210, R^2^ = 0.996 (y is the R ratio, and x is the total protein amount) (Fig. 1A b). Thus, we verified that agarose gel electrophoresis could be used to detect enzymatic activity. The endonuclease activities of HO8910 cells at various numbers (2 × 10^5^, 1 × 10^5^, 8 × 10^4^, 4 × 10^4^, and 2 × 10^4^) in 100 μl Chaps lysis buffer were detected by two different methods: agarose gel electrophoresis (Fig. 1B a), and colony counting of bacterial clones (Fig. 1B c). The endonuclease activities of HO8910 cells at various numbers (2 × 10^5^, 1 × 10^5^, 8 × 10^4^, 4 × 10^4^, and 2 × 10^4^) in 100 μl Chaps lysis buffer were quantitatively analyzed by Image J (R ratios = 2.30155, 1.577371, 1.429271, 1.274542, and 1.263294, respectively) (Fig. 1B b).Bacterial colony number histograms of HO8910 cells showed that the lower the numbers of HO8910 cells, the higher the number of colonies (Fig. 1B d). The number of colonies was inversely proportional to the degree of plasmid cleavage. The same conclusion was reached by the colony count of bacterial clones and agarose gel electrophoresis. Therefore the feasibility of detecting the activity of endonuclease by agarose gel electrophoresis was verified.

### Optimal experimental conditions of agarose gel electrophoresis

**Fig. 2:**
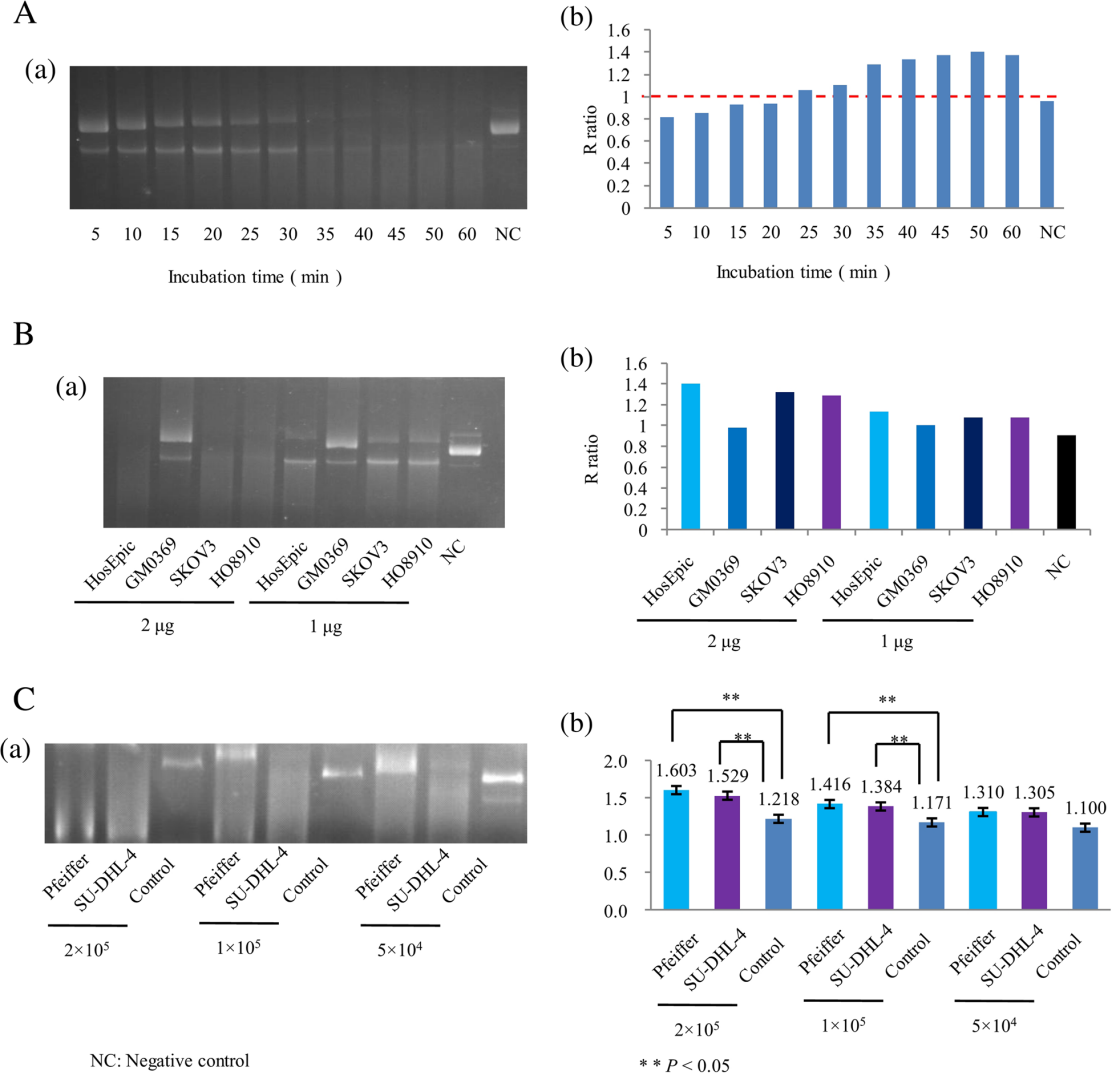
Optimization of the incubation time and number of cells in agarose gel electrophoresis. **A** (a) Agarose gel electrophoresis of HO8910 cell with different incubation times. (b) Histogram of R ratios with different incubation time. **B** (a) Agarose gel electrophoresis of different cells with different total protein amounts. (b) Histogram of R ratios with different cell and total protein amounts. **C** (a) Agarose gel electrophoresis of lymphoma cell lines (Pfeiffer and SU-DHL-4) and normal controls. Lanes 1,4, and 7: Pfeiffer cells; Lanes 2,5, and 8: SU-DHL-4 cells; Lanes 3,6, and 9: normal control. Lanes 1–3: 2 × 10^5^ cells; Lanes 4–6: 1 × 10^5^ cells; Lanes 79: 5 × 10^4^ cells. (b) Histogram of R ratios of lymphoma cell lines and normal controls

Different incubation times caused different plasmid cleavages (Fig. 2A a). Figure 2A b shows a histogram of R ratios with different incubation times. If the incubation time was too short, the R ratio could be too low to distinguish. The optimal incubation time was 30 min to avoid blurry bands in agarose gel electrophoresis.

Different cell numbers and total protein also caused different plasmid cleavages (Fig. 2B a). The lysates obtained from different cell numbers had different degrees of cleavage and enzymatic activity at the same total amount of protein. Figure 2B b shows a histogram of R ratios at different cell numbers and total amounts of protein.

Lymphoma cell lines Pfeiffer and SU-DHL-4, and normal controls at various numbers (2 × 10^5^, 1 × 10^5^, and 5 × 10^4^) were applied to agarose gel electrophoresis to determine the R ratios (Fig. 2C a). The R ratios of lymphoma cell lines (Pfeiffer and SU-DHL-4) were higher than that of normal controls. Figure 2C b shows a histogram of the R ratios of lymphoma cell lines and normal controls. At 2 × 10^5^ or 1 × 10^5^ cells, the R ratio of Pfeiffer cells was higher than that of the control (*P* = 0.020 and *P* = 0.024, respectively). There were also statistically significant differences in the R ratio of SU-DHL-4 cells and the control (*P* = 0.044 and *P* = 0.040, respectively). This result indicated that the endonuclease activity of lymphoma cells was higher than that of normal cells. In addition, as the number of cells decreased, the difference became smaller. At 5 × 10^4^ cells, there was no statistically significant differences in the R ratios between of Pfeiffer and control cells (*P* = 0.090) or SU-DHL-4 and control cells (*P* = 0.097). This result suggested that the experimental specimen required a certain number of cells.

The optimal electrophoresis procedure for mononuclear cells was as follows. A total of 2 × 10^6^ mononuclear cells was collected from each specimen, and then 100 μl lysate was incubated in an ice bath for 60 min, followed by centrifugation at 2500×*g* for 10 min to extract the supernatant. The lysate supernatant was stored at − 80 °C until use. A total of 200 ng *pwpxl* was added to 15 μl supernatant, followed by H_2_O to adjust the total volume to 20 μl. After incubation at 37 °C for 30 min, 4 μl of 6 × loading buffer was added, followed by mixing. A 1% agarose gel was prepared, and 20 μl of mixture was applied to each lane. Finally, 130 V electrophoresis was performed for 1 h.

### Degree of plasmid digestion by tissue lysate

**Fig. 3:**
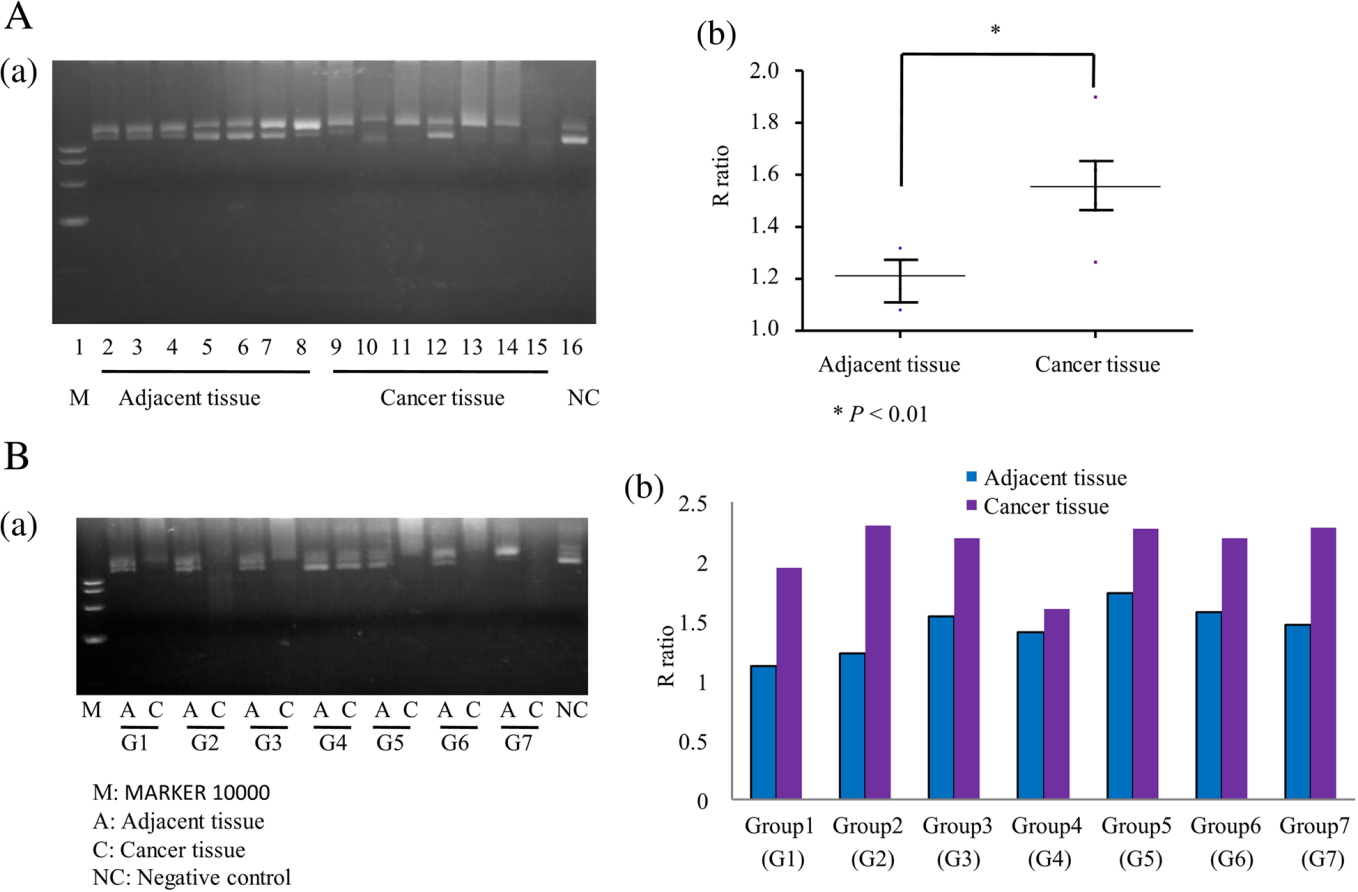
Degree of plasmid digestion by tissue lysates. **A** (a) Agarose gel electrophoresis of cancer and adjacent tissues. Lane 1: MARKER 10000, lanes 2–8: adjacent tissue, lanes 9–15: cancer tissue, lane 16: negative control. (b) R ratios in the adjacent tissue group compared with the cancer tissue group. **B** (a) Agarose gel electrophoresis of the same patient’s cancer tissue and matched adjacent tissue. Lane 1: MARKER 10000, lane 2: adjacent tissue, lane 3: matched cancer tissue from the same patient in lane 2, lane 4: adjacent tissue, lane 5: matched cancer tissue from the same patient in lane 4, lane 6: adjacent tissue, lane 7: matched cancer tissue from the same patient in lane 6,…, lane 14: adjacent tissue, lane 15: matched cancer tissue from the same patient in lane 14, and lane 16: negative control. (b)R ratios of the same patient’s cancer and adjacent tissues

The endonuclease activities of cancer and adjacent tissues in ovarian cancer patients(*n* = 7) were detected by agarose gel electrophoresis. As shown in Fig. 3A a, lane 1 is MARKER 10000, lanes 2–8 are adjacent tissue, lanes 9–15 are cancer tissue, and lane 16 is the negative control. There was a significant difference in endonuclease activities of adjacent and the cancer tissues (*P* = 0.001) (Fig. 3A b).

The endonuclease activities of the same ovarian cancer patient’s cancer tissue and matched adjacent tissue (n = 7)were detected by agarose gel electrophoresis. As shown in Fig. 3B a, lane 1 is MARKER 10000, lane 16 is the negative control, lanes 2 and 3 are the adjacent tissue and cancer tissue, respectively, from the same patient, and lanes 4 and 5 are another patient’s adjacent tissue and cancer tissue. Figure 3B b shows a histogram of R ratios of the same patient’s adjacent and cancer tissues. There was a significant difference in endonuclease activities of the same patient’s adjacent and cancer tissues (*P* < 0.001).

### Degree of plasmid digestion by mononuclear cell lysates

**Fig. 4:**
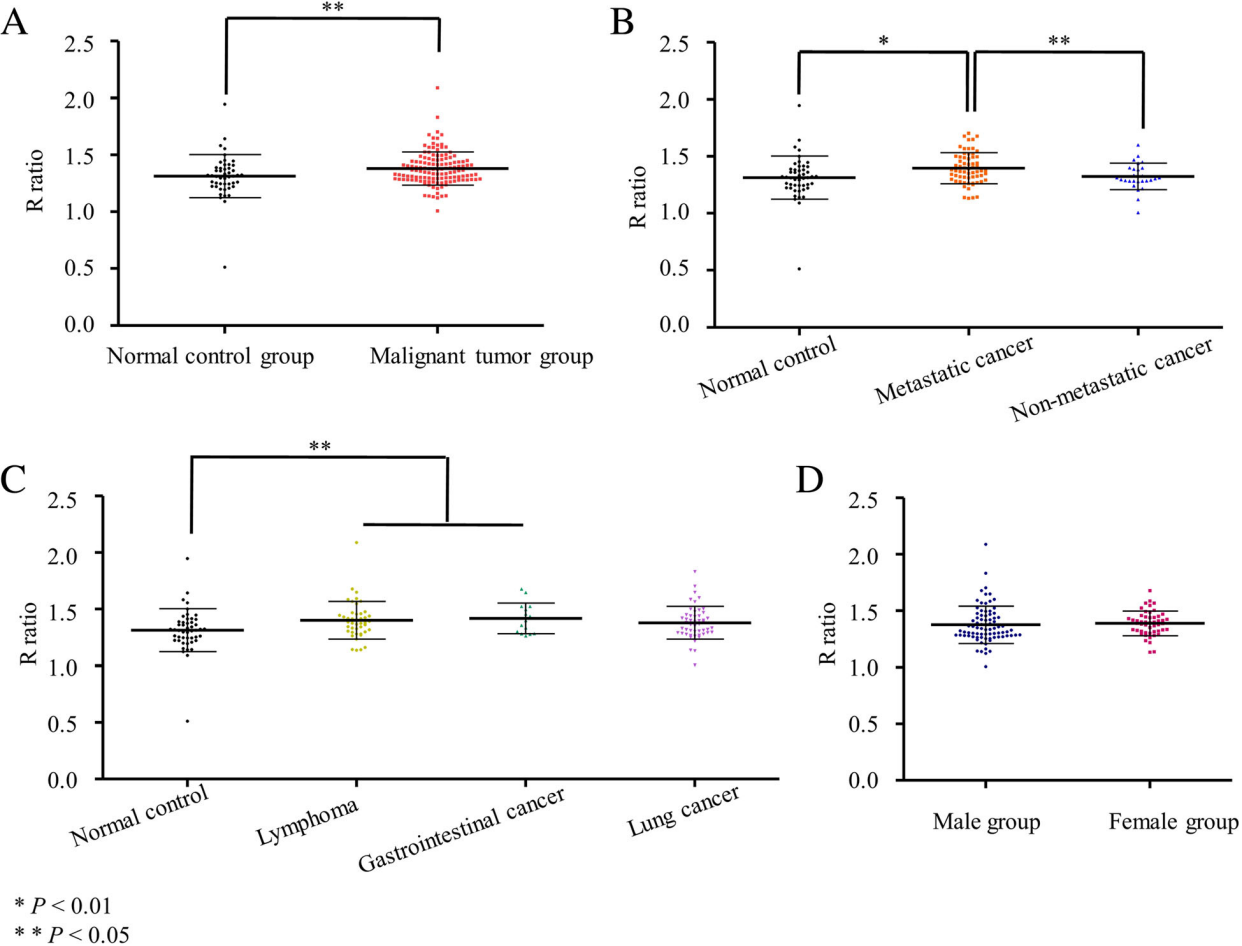
Degree of *pwpxl* digestion by mononuclear cells lysate. **a** R ratios in the normal control group compared with the malignant tumor group. **b** R ratios in normal control, metastatic cancer and non-metastatic cancer groups. **c** R ratios in normal control, malignant lymphoma, gastrointestinal cancer, lung cancer groups. **d** R ratios in the male cancer group compared with the female cancer group

The experimental conditions were as follows: 2 × 10^6^ mononuclear cells in 100 μl lysis buffer, 15 μl lysate, 200 ng *pwpxl*, 1% agarose gel, and electrophoresis for 1 h at 130 V. The activity of endonuclease was also much higher in the malignant group (*n* = 131) than in the control group (*P* = 0.015) (Fig. 4a). In addition, there were significant differences between the normal control group (*n* = 48) and malignant lymphoma group (*P* = 0.013), and normal control and gastrointestinal cancer groups (*P* = 0.036) (Fig. 4c). However, there was no significant difference in endonuclease activities of control and lung cancer groups (*P* = 0.056). There was no significant difference in endonuclease activities of the malignant lymphoma group (*n* = 44), gastrointestinal cancer group (*n* = 15) and lung cancer group (*n* = 50).

There was a significant difference in the endonuclease activities of metastatic cancer group (*n* = 64) and non-metastatic cancer group (*n* = 28) (*P* = 0.038), and metastatic cancer group and normal control group (*P* = 0.006) (Fig. 4b). However, there was no significant difference in the endonuclease activities of control and non-metastatic cancer group (*P* = 0.800) (Fig. 4b). There was no significant difference between male and females in the normal group (Fig. 4d), or cancer group (*P* = 0.564 and *P* = 0.635, respectively).

### Prognostic significance assessed by PFS analysis

**Fig. 5:**
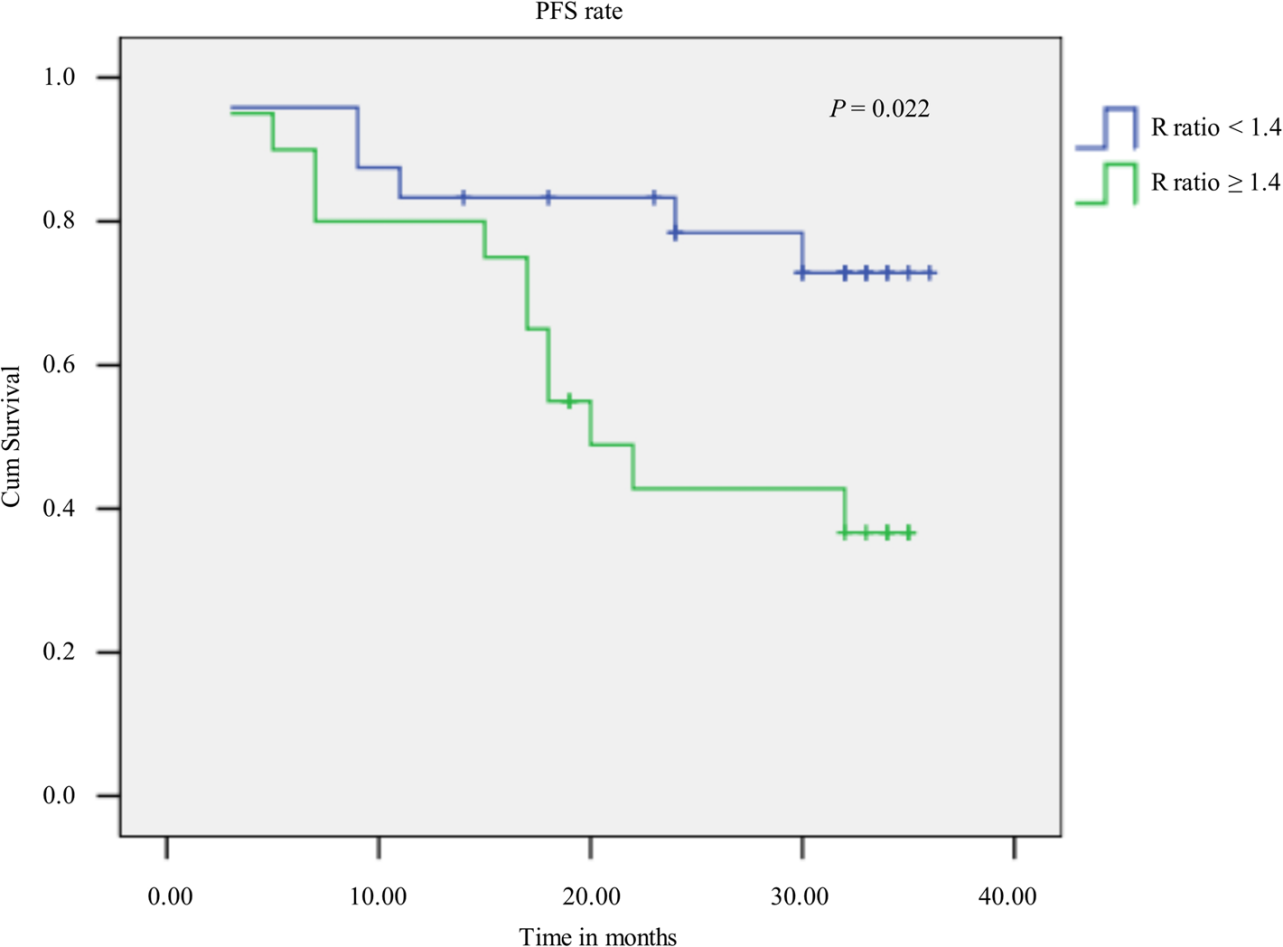
Kaplan-Meier progression-free survival curve of patients. Patients with elevated R ratios had significantly lower PFS than patients with lower R ratios. PFS, progression-free survival

We further investigated the significance of the R ratio in the prognosis of lymphoma patients. Among 44 patients with malignant lymphoma, the outcomes of patients with elevated R ratios (R ratio ≥ 1.4) were the poorer than those of patients with lower R ratios (R ratio < 1.4) (*P* = 0.022; 95% confidence interval: 17.64–27.77) in Kaplan-Meier survival analysis, indicating that patients with an R ratio of ≥1.4 patients have a clinical disadvantage (Fig. 5).

## Discussion

In preliminary-experiments, there was a significant difference in endonuclease activities of adjacent and cancer tissues (*P* < 0.001). However, tissue sampling is traumatic, and the patient’s medical performance will be poor. Endonuclease activity in cancer tissue was higher than that in adjacent tissues of patients. Therefore, the feasibility of the experimental method was confirmed in this study. Agarose gel electrophoresis and the colony count of bacterial clones to identify the different amount of 8910 cells between groups led to the same results, verifying the feasibility of agarose gel electrophoresis detection of endonuclease activity.

The endonuclease activities of control and malignant tumor groups were significantly higher than that of the control group (*P* = 0.015). In addition, there was a significant difference between normal control and malignant lymphoma groups (*P* = 0.013), and normal control and gastrointestinal cancer groups (*P* = 0.036). However, there was no significant difference in the endonuclease activities of control and lung cancer groups (*P* = 0.056). The median, quarter median, 3rd quartile, mean and 95% confidence interval of the lung cancer group were all higher than those of the normal control group, which may be caused by the small number of experiments. These data suggest that endonuclease activity might be a potential tumor marker.

There was a significant difference in the endonuclease activities of metastatic cancer and non-metastatic cancer groups (*P* = 0.038). The endonuclease activity was significantly higher in the metastatic cancer group than in the control group (*P* = 0.006). This result demonstrated that endonuclease activity was associated with the malignancy of malignant tumors. In this study, we did not find strong evidence to support the use of endonuclease as a prognostic factor. There are some studies with conclusions similar to our study. Woo et al. suggested that APE1 expression may be associated with breast cancer prognosis. In particular, its role as a prognostic factor may be significant for breast cancers with a low proliferation index [16]. Shin et al. [17] found that telomerase activity was significantly higher in peripheral blood of patients with gastric cancer than in non-tumor patients, which was closely related to distant metastasis. There are reports regarding patients with lymphatic metastasis having a poor prognosis. For example, lymphatic metastasis is recognized as an early adverse event in progression of pancreatic cancer and has been described as an independent poor prognostic factor [18]. Because most tumor treatment failures are due to metastasis, endonuclease activity has the potential to be a prognostic factor. There are some reports about the relationship between prognosis and endonucleases including APE1, FEN1, and Dicer. For example, most studies that determined whether APE1 can be used as a predictive marker showed that high level expression or cytoplasmic staining were associated with poor outcomes and resistance to chemoradiotherapy in patients with lung cancer, breast cancer, head and neck cancer, osteosarcomas, germ cell tumors and hepatocellular carcinomas [19]. Jiao et al. revealed that FEN1 was associated with the risk of gallbladder cancer [20]. Dicer expression might also be an indicator of prognosis for chronic lymphocytic leukemia [21].

However, there was no significant difference in the endonuclease activities of control and non-metastatic cancer groups (*P* = 0.800).The rapid proliferation of tumor cells depends on the continuous expansion of microvascular networks, accompanied by angiogenesis and rapid focal growth. The greater the probability that cancer cells enter blood circulation, the greater the probability of tumor metastasis [22]. There were no differences between control and non-metastatic cancer groups which may be due to fewer cancer cells entering peripheral blood in non-metastatic cancer patients. Endonuclease activity has the potential ability to be a metastasis factor. However, we can not exclude that the sample size in our study was too small to detect a significant effect.

In a previous study, other DNA cleavage enzymes such as Dicer with abnormal expression had been shown in various cancers including some subtypes of T cell lymphoma, which influenced the patient prognosis [23]. In this study, the R ratio of lymphoma cell lines (Pfeiffer and SU-DHL-4) was higher than that of normal controls. Furthermore, the R ratio of the lymphoma group was higher than that of normal control group (*P* = 0.013). Although there was no significant difference between the control group and non-metastatic cancer patients (*P* = 0.800 > 0.05), endonuclease activities were lower in the control group compared with non-metastatic lymphoma patients (*P* = 0.033). This result suggested that endonuclease activity might be a potential tumor marker of lymphoma. Furthermore, our results revealed that patients with higher R ratios had significantly shorter PFS than those with lower R ratios. Therefore, our study indicated that endonuclease activity could be a potential marker for screening and prognosis of malignant lymphoma.

Preliminary experiments showed that liver cancer, ovarian cancer, and normal control groups had no significant difference in endonuclease activity (data not shown). This result may be due to liver cancer patients with liver dysfunction affecting endonuclease activity. The liver is the main site of protein and enzyme synthesis, and a variety of protein concentrations and enzymatic activities change during liver cirrhosis.

There was no significant difference in endonuclease activities of different kinds of cancer. To distinguish between different cancer types by detecting endonuclease activity, it is necessary to further explore the difference in the content of the various types of endonucleases between different tumors. In summary, the elevated activity of endonucleases may be associated with cancer metastasis. Our study suggests that detection of endonuclease activity might be a potential method for prognosis prediction of cancers, especially lymphoma.

## Conclusions

In this study, we established a method to detect the DNA endonuclease activity and found an association of endonuclease activity with prognosis, especially inmalignant lymphoma. This finding suggests that this detection assay for endonuclease activity is useful to predict the prognosis of malignant lymphoma.

## Abbreviations

APE1: Apurinic/apyrimidinic endonuclease 1
CLL: Chronic lymphocytic leukemia
EDTA: Ethylenediaminetetraacetic acid
FEN1: Flap endonuclease-1
HL: Hodgkin
LP-BER: Long Patch Base Excision Repair
NC: Negative control
NHL: Non-Hodgkin
PFS: Progression-free survival
